# Effects of Wood Particles from Deadwood on the Properties and Formaldehyde Emission of Particleboards

**DOI:** 10.3390/polym14173535

**Published:** 2022-08-28

**Authors:** Pavlo Bekhta, Ruslan Kozak, Vladimír Gryc, Václav Sebera, Jan Tippner

**Affiliations:** 1 Department of Wood-Based Composites, Cellulose and Paper, Ukrainian National Forestry University, 79057 Lviv, Ukraine; 2 Department of Wood Science and Technology, Faculty of Forestry and Wood Technology, Mendel University in Brno, Zemědělská 3, 613 00 Brno, Czech Republic

**Keywords:** particleboards, deadwood, wood particles, formaldehyde emission, urea-formaldehyde adhesive, bending strength, internal bond strength, modulus of elasticity, thickness swelling

## Abstract

The volume of deadwood increases annually because of changes in environmental, climatic, and hydrological conditions. On the other hand, during the last decade, manufacturers of wood-based boards have been facing an acute problem of a shortage of conventional raw materials. The purpose of this study was to evaluate the possibility of using wood particles from deadwood in the production of particleboards. Three-layer particleboards with different content of deadwood particles (0%, 25%, 50%, 75%, 100%) were produced. Conventional urea-formaldehyde (UF) resin was used for gluing the particles. The physical and mechanical properties of the boards, as well as the formaldehyde content in the boards, were determined. In addition, the effect of adding melamine-urea-formaldehyde (MUF) resin to UF adhesive on the properties of the boards was investigated. Replacing conventional sound wood particles with deadwood particles leads to deterioration of the physical and mechanical properties of the boards. The boards from deadwood particles absorb more water and swell more. The bending strength (MOR), modulus of elasticity in bending (MOE), and internal bonding (IB) values for boards with 100% deadwood particles are reduced by 26.5%, 23.1%, and 72.4%, respectively, compared to reference boards from sound wood particles. Despite this, a significant advantage is that boards made from 100% deadwood particles are characterized by 34.5% less formaldehyde content than reference boards made from conventional sound wood. Moreover, adding 3% of MUF resin to UF adhesive increases MOR, MOE, and IB by 44.1%, 43.3%, and 294.4%, respectively.

## 1. Introduction

**Figure 1:**
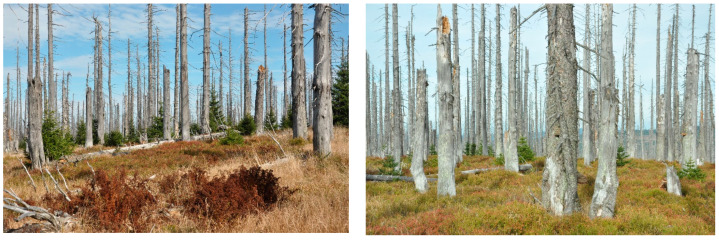
Dead trees in Šumava National Park, Czech Republic (Ing. Tomáš Koutecký, Ph.D).

**Figure 2:**
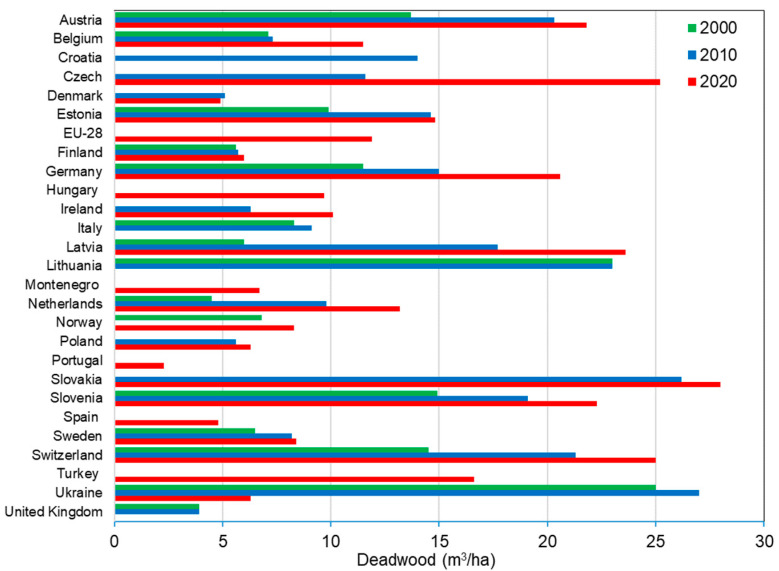
Average volumes of deadwood in European countries, 2000–2020 [2,3].

An intensive consumption of wood has led to a shortage of wood of industrial value in many countries of the world. This encourages wood processing firms to search for additional wood resources suitable for industrial use. Deadwood (dead fallen and standing trees, as well as felled by a windstorm etc.) (Figure 1) can be such an unused resources of raw materials. Nowadays, the challenge of tree drying in the forests is one of the acute ones for the whole of Europe. An increase in the average annual temperature and a decrease in the average annual precipitation contribute to the rapid reproduction of fungal diseases, various bacteria and pests and the growth of stocks of deadwood. Millions of cubic meters of infested trees (beetle-killed trees are very common) are now standing in Europe forests (Figure 1). With such rapid climate changes observed in recent years, the area of such deadwood can be expected to increase. Currently, the major European forest product companies harvest green wood. However, it is possible that in the near future, the majority of available wood will be infested wood. In unmanaged Central European forests, deadwood usually comprises up to 25% of the entire volume of wood in the forest [1]. Over the last 25 years, the amount of deadwood has increased in all European regions (Figure 2) [2,3]. This could be explained by more frequent disturbances such as storms, insects’ outbreaks and forest fires caused also by changing climatic conditions. It seems that temperature and moisture are the driving factors. The average volume of deadwood in 2015 is above 11 m^3^/ha, equal to above 7% of the average volume of the growing stock density of European forests [2,3]. From Figure 2, it can be seen that in countries with mountain forests, the amount of deadwood is 2–3 times higher than in lowlands.

We have to remember that deadwood is a crucial component of forest ecosystems and plays an essential role in sustaining biodiversity as well as in processes such as soil formation and nutrient cycling [4]. However, we cannot also forget that wood is an important renewable commercial commodity, irreplaceable in numerous applications necessary for society’s development [2]. Therefore, considering that deadwood represents a significant economic resource, economic uses of this resource need to be carefully considered [5].

Over the years, research efforts have focused on the study of how well deadwood is suited for the manufacture of a range of products (power poles, pallets, round timbers, lumber, laminated wood, panels, pulp and paper, fuel etc.) and to better understand how such wood will affect different products. Despite that, the use of deadwood is currently limited. This is often because certain challenges exist through all phases of the production of wood products from deadwood, including harvesting, transportation, log storage, processing, and end-product marketing [6].

Woo et al. [7] found that infested wood compared to sound wood is characterized by substantial loss of moisture, lower density, significantly lower concentrations of extractives, lower lignin and hemicellulose contents and better permeability. Another authors [8] noted that standing dead trees lost a 50% more of moisture content compared to fallen trees or trees on the ground. Such excessive dryness of deadwood creates technical problems for its use [5]. For example, the reduction in the moisture content of the wood makes it prone to checking and warping. This negatively affects the value of lumber and chip quality for pulping [7,9], as well as causes problems with the quality of flakes (or strands) used in composite boards and with maintenance of cutting tools [10]. However, on the other hand, as deadwood dries, it becomes lighter, which reduces transportation costs, and requires less drying time, which can save production costs [5].

Increased permeability of deadwood indicates possible irregular absorption or over-absorption of finishes and glues [11]. Troxell et al. [12] observed an increase of up to three times in chemical uptake when deadwood is considered.

Wood density decreases significantly over the course of decomposition [7,13,14,15]. For example, while wood density of living Norway spruce trees is about 0.43 g/cm^3^ [13], the average density of the most decayed Norway spruce deadwood is only 0.138 g/cm^3^ [14]. Other authors mentioned similar results. At one year after death, the density of beetle-killed southern pine was 60% of green timber [15].

According to the literature, the influence of different types of pests on the strength of wood is unclear. Some authors observed a reduction of 30–40% in toughness, of 11% in stiffness or modulus of elasticity, and of 19% in breaking strength or modulus of rupture in southern pine beetle-killed timber [16]. Walters [17] pointed out that southern pines had shown a reduction of 12% in bending strength (MOR) and 13% in modulus of elasticity in bending (MOE) after one year since death. On the contrary, several authors showed that infested wood has quite similar properties (including strength characteristics) to those of green timber [18] or has no effect on wood mechanical properties [19].

In the deadwood, because of the action of biologically active organisms, in addition to changes in physical and mechanical parameters, there are also changes in the chemical composition. Seifert [20] found losses of 7% cellulose and 3–4% hemicellulose in blue-stained timber, which could be related to changes in permeability and/or toughness. Thus, with a deep degree of mycological destruction, the physical and mechanical properties of wood deteriorate to such an extent that it becomes unsuitable for use as a construction material. However, the morphological structure and chemical composition allow its use as an active filler for the manufacture of wood composite materials.

Several authors [21] concluded that dead pines after an outbreak is a suitable feedstock for the production of lignocellulose micro-/nanofibrils. They found that the tree with advanced decay, which has no value for lumber, produced lignocellulose nanofibrils similar to those from the live tree. Studies also found that the infested trees have great potential for wood-plastic composites [22,23] and cement-bonded particleboard [24].

Various researchers have studied the possibility of using infested wood to make veneers and plywood. The most serious problems that were observed when processing dead wood into veneer were reduced veneer yield and reduction in full-sheet recovery. For example, Walters and Weldon [25] found a 9% less veneer volume, fewer full sheets and a higher percentage of random-width veneer, whereas Snellgrove and Ernst [26] found a 30% reduction in volume recovery and a higher percentage of random-width veneer for wood after three years since tree death. On the contrary, several authors found that there was no significant difference in veneer recovery between green and dead timber, especially when the affected trees are used immediately after the attack [25]. To improve veneer recovery from beetle-killed logs, some authors recommend proper conditioning of the logs [27]. They also showed that beetle-wood veneer can be dried faster, with a reduction in drying time by about 35% and a 27% increase in productivity from veneer drying.

Some authors note that the costs are similar in making particleboards from beetle-killed timber and from green trees, and significant equipment modifications for the production of such boards (using deadwood) is not required [28]. Moreover, studies showed an improved quality of particleboard when adding blue-stained timber into the furnish [29]. However, on the other hand, the use of beetle-killed wood leads to an increase in the amount of small fine fraction produced, the need for extra screening capacity and additional extra maintenance for cutting knives, the decrease in slenderness, and the tendency of the flakes to become folded [28,30].

A positive aspect regarding the use of deadwood in the production of particleboards is its significantly lower cost and limited application currently, compared to the traditional sound wood, which makes such a raw material attractive from an economic point of view. A negative aspect is the reduction of physical and mechanical properties of wood composites made from deadwood. It would be possible to compensate the loss in mechanical properties of composites from deadwood by using more reactive adhesive compositions than those traditionally used. Urea-formaldehyde (UF) resins are the most widely used adhesives in the manufacture of particleboards. In practice, melamine-urea-formaldehyde (MUF) resins are often added to UF adhesives to improve its adhesive strength and water resistance properties [31]. However, the modification of UF adhesives with MUF resin for the production of particleboards from deadwood requires additional research. Moreover, the UF adhesives have a major drawback, connected to the hazardous emission of volatile organic compounds (VOCs) and free formaldehyde from the finished particleboards [32]. As a result, new formaldehyde emission restrictions have been set for wood-based composites in Europe, the United States, and Japan. From the other hand, it was found that the emission of volatile organic compounds from wood decreases with wood storage tremendously. The emission of VOC from pine wood decreased by 50% on storage for 14 days [33]. Furthermore, Schäfer and Roffael [34] found that with increasing storage time, the spruce and pine particles emit less formaldehyde than non-stored wood. Based on this, we can assume that the addition of deadwood to the traditional wood in the production of particleboards can reduce the formaldehyde emission of the boards.

As follows from the literature resources, the general strategy would be to use deadwood as soon after death as possible, because the longer it’s dead, the more it deteriorates, and the fewer are the options for its utilization [35]. Increasing amounts of deadwood and related literature provide insights into the feasibility of converting deadwood into composite wood panel products including particleboard. However, the use of deadwood for particleboards will not be possible without a comprehensive knowledge of the physical and chemical characteristics of this wood and the impact that these characteristics would have on a manufactured board’s properties [6].

Thus, the objective of the present study was to evaluate the possibility of using wood particles from deadwood in the production of particleboards and to find out how the amount of deadwood particles affect the physical and mechanical properties, as well as the formaldehyde content of the boards.

## 2. Materials and Methods

### 2.1. Materials

Factory-produced wood particles from deadwood and traditional (sound) wood comprised of coniferous (75%) and deciduous (25%) species (originated from the Ukrainian Carpathians, Ivano-Frankivsk region) were obtained from the local particleboard plant. The deadwood was stored in the raw material warehouse for approximately four months prior to processing. Pine (*Pinus sylvestris* L.) and beech (*Fagus sylvatica* L.) woods mostly prevail among conifers and deciduous species, respectively. The moisture content of the particles, determined by the drying-weighing method, was approximately 3%. The fractional composition of the particles from deadwood and sound wood for the outer and core layers of the boards is presented in Table 1.

UF and MUF resins were used in the experiments. UF adhesive consisted UF resin grade A (density 1.28 g/cm^3^, solid content 66%, Ford cup (4 mm, 20 °C) viscosity 98 s, pH = 7.8, gel time 50 s) (producer LLC “Karpatsmoly”, Kalush, Ukraine), paraffin emulsion, urea, and ammonium sulfate. A 33% aqueous solution of ammonium sulfate [(NH_4_)_2_SO_4_] was used as a hardener and was mixed with the UF resin before spraying onto the wood particles. A 43% aqueous solution of urea [CO(NH_2_)_2_] and paraffin emulsion were mixed with UF resin.

MUF resin (density 1.29 g/cm^3^, solid content 64.3%, viscosity 224 (Brookfield)/41 KF, pH = 9.32, gel time 83 s), due to its high reactivity towards wood surface and UF resin molecules, was used as an additional component to UF adhesive to improve water resistance and mechanical properties of particleboards. To find out how the addition of MUF resin to UF adhesive affects the properties of particleboards manufactured from deadwood, 1% and 3% of MUF were added to UF adhesive.

### 2.2. Manufacture of Particleboards

**Figure 3:**
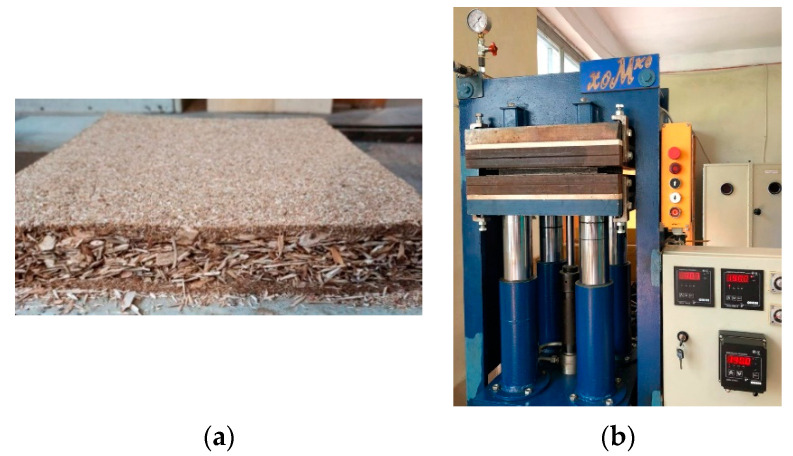
The formed particle mat (**a**) and hydraulic laboratory press (**b**).

Three-layered particleboards of 290 × 290 mm dimensions and a thickness of 16 mm with a target density of 650 kg/m^3^ were made. The boards contained particles from deadwood and sound wood. Particles from deadwood were added to the outer and core layers of the boards in the amount of 25%, 50%, 75% and 100%. The mass share of the outer layers was 33%, and the core layer was 67%. The amounts of UF resin, urea, hardener, and paraffin emulsion that were required for the mixing process were different for the core layer and the outer layers. This is due to the temperature difference between the surface and the core caused by heat transfer from the surface to the core of the board. In addition, the different amount of resin and additives used is related to the difference in the surface area of the particles used in the core and outer layers of the board. The amount of solid UF resin was 14 wt.% and 9 wt.% based on the weight of oven-dried wood particles for the outer and middle layers, respectively. During resin mixing, 2.3% and 0.5% of urea solution and 0.2% and 0.6% of ammonium sulfate were added, based on the weight of dry particles, to UF resin for the outer and core layers, respectively. A 0.8% of paraffin emulsion based on the weight of dry particles was also included in the resin mixture. The MUF resin was added to the UF adhesive used for the core layer. Wood particles were mixed with adhesive by hand. After mixing, the resinated particles were evenly distributed by hand in a 290 mm × 290 mm rectangular wooden mold. Pre-pressing of the formed mat was carried out manually in the wooden box. Next, the mat (Figure 3a) was subjected to hot pressing in an automatically controlled hydraulic laboratory press “xoMкo” (LLC “ODEK” Ukraine, Ukraine) (Figure 3b) at the pressure of 2.5 MPa, temperature of 190 °C and the pressing time of 22.5 s/mm. During the last 30 s of the pressing cycle, the pressure was continuously reduced to 0 MPa. The experimental design for this study is summarized in Table 2.

### 2.3. Particleboards Testing

After pressing, the boards were stored in air until reaching room temperature. Then, before evaluating their properties, the boards were conditioned for one week in a conditioning room, where the relative humidity of 65 ± 5% and 20 °C were maintained. The moisture content of the boards was within 6%. Three boards were made for each type of particleboard in the experimental design (Table 2), i.e., 21 boards. The conditioned boards were cut into required testing size according to relevant standards. Three samples of each board were tested according to European standards for density (EN 323) [36], bending strength (EN 310) [37], modulus of elasticity in bending (EN 310) [37], internal bond (IB) strength (EN 319) [38], thickness swelling (TS) (EN 317) [39] and water absorption (WA). On the other hand, for each batch, one board was randomly selected for analysis of formaldehyde content (FC) based on EN ISO 12460-5 (perforator method) [40].

The effects of wood particles content from deadwood and the amount of MUF resin on the properties of the laboratory-made boards was evaluated using an analysis of variance (ANOVA) at a significance level of 0.05. Duncan’s range tests were performed to determine significant differences between means.

## 3. Results

In this study, a great difference in the amount of fine fractions of particles between deadwood and sound wood was not observed (Table 1). However, other authors indicate that logs dried to an average 50% moisture content produced nearly double the fines relative to green logs [41]. Fractional analysis of the wood particles (Table 1) used in this study showed that more fine particles prevailed in the particles obtained from deadwood. This can be considered as a factor that can significantly affect the properties of the boards. It is generally known that the quality of wood particles is the key factor in limiting production of quality particleboard as the geometry of particles affects the board’s physical properties and internal bond strength characteristics [28,30].

### 3.1. Physical Properties of Boards

Table 3 presents mean values of density, WA and TS after 2 and 24 h immersion in the water for boards manufactured with adding different amount of deadwood particles and various amount of MUF resin into the UF adhesive. Deviations of the average values of the densities of the boards from the target density of 650 kg/m^3^ are caused by the effect of material loss during the formation of the carpet, the uneven laying of wood particles over the area of the carpet, as well as the manual formation itself. However, these deviations of 1.2–5.3% were only marginally significant and did not affect significantly the results of the board’s property values.

**Figure 4:**
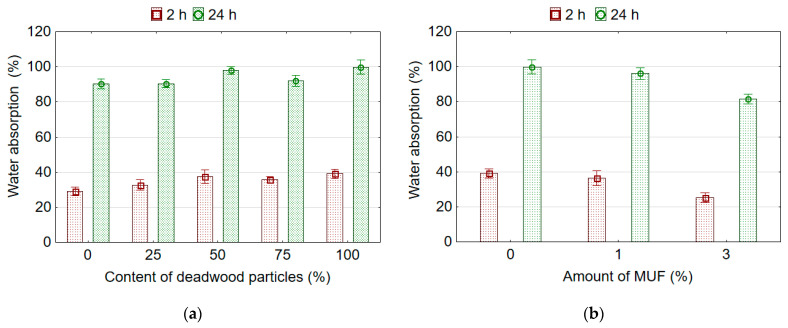
Water absorption of boards samples depending on content of deadwood particles (**a**) and amount of MUF resin in adhesive (**b**).

It was found that wood particles content from deadwood and the amount of MUF resin added into the UF adhesive have a significant effect on the TS and WA of the boards after soaking in water for 2 and 24 h. It can be stated that the influence of wood particles content from deadwood was less pronounced than the influence of the amount of MUF resin. Replacing sound wood particles with deadwood particles results in increased WA after 2 and 24 h of soaking in water. The lowest values of WA 2 h (29.01%) and WA 24 h (90.24%) were observed for boards made from sound wood particles. The highest WA after 2 h (38.96%) and 24 h (99.76%) of soaking in water was observed in the boards made with 100% deadwood particles. In addition, as can be seen (Figure 4a), more than a third of all the water absorbed by the samples is absorbed by the samples during the first 2 h. In addition, within the first 2 h, samples from sound wood particles (type A) absorb 32.1% of water, and samples from deadwood particles (type E) absorb 7% more water (39.1%).

**Figure 5:**
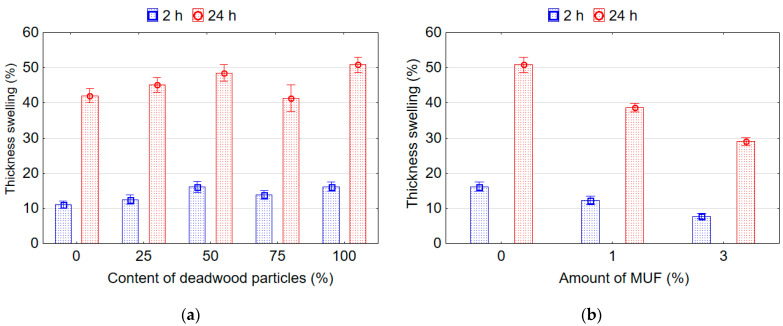
Thickness swelling of boards samples depending on content of deadwood particles (**a**) and amount of MUF resin in adhesive (**b**).

A similar trend is observed for TS. The presence of deadwood particles in the boards negatively affects the indicators of its swelling in water (Figure 5a). The samples with the addition of deadwood particles swell more than samples made from conventional sound wood particles. The lowest TS 2 h value 11.03% was found for the boards made of sound wood particles, and the highest value 16.17% for the boards made of deadwood particles. The lowest TS 24 h values were found for the boards with 75% deadwood particles (41.36%) and conventional sound wood particles (42.01%), and the highest 48.55% and 50.82%, respectively, in boards with 50% and 100% deadwood particles.

The boards with 25% (type B) and 75% (type D) of deadwood particles in terms of WA 2 h, WA 24 h, TS 2 h and TS 24 h do not differ significantly from each other and reference boards (except WA 2 h) made from conventional sound wood particles (type A). In contrast, the values of WA 2 h and TS 2 h for the boards D differ significantly from those values for the reference boards (type A). Likewise, the WA 2 h, WA 24 h, TS 2 h and TS 24 h of the boards with 50% (C) and 100% (E) deadwood particles do not differ from each other but differ significantly from the reference boards made from conventional sound wood particles (type A).

The addition of MUF resin into UF adhesive reduces the WA and TS of board samples made from deadwood particles. With an increase in the amount of MUF in the adhesive up to 3%, the values of WA 2 h, WA 24 h, TS 2 h, and TS 24 h compared to the adhesive without the addition of MUF resin decrease by 35.1%, 18.2%, 52.2%, and 42.9%, respectively.

Similar results were obtained by other authors who indicate that the oriented strand boards (OSB) derived from 100% mountain pine beetle-killed wood (standing dead for 2 or for 20 years) had greatly reduced water-resistance properties and dimensional stability [5]. The increase in WA and TS of boards containing deadwood particles is caused by the fact that the deadwood particles, due to destructive changes in its structure, have a large number of cracks that are formed during chipping. Water penetrates through the cracks in the board structure, destroys the UF adhesive joints, causes swelling of not only particles in outer layers, but also particles in the core layer, and fills additional voids that are formed because of destructive processes. The obtained results and their explanations are in good agreement with the results of other authors. For example, several authors found as much as 14% of the density losses due to decay for white pine with deep checks and no twigs, which is a sign of the loss of wood substance and the increase of its porosity [30]. In contrast, other authors mentioned that at one year after death, the density of beetle-killed southern pine was 60% of green timber [15]. In addition, increased permeability of wood particles from deadwood allows for greater water penetration [42]. These authors [42] suggest that the mechanism for increased permeability is probably the opening up of ray parenchyma cells by blue-stain fungi, and the microcracking that could be observed on some lumber samples.

The adding of water-resistant MUF resin into UF adhesive compositions allows reducing the negative impact of water on the values WA and TS of the boards containing deadwood particles. Adhesive bonds are destroyed less, which makes it difficult for water to penetrate the core of the board and causes a decrease in the swelling of the particles in the core layer and the formation of additional voids.

### 3.2. Mechanical Properties of Boards

**Figure 6:**
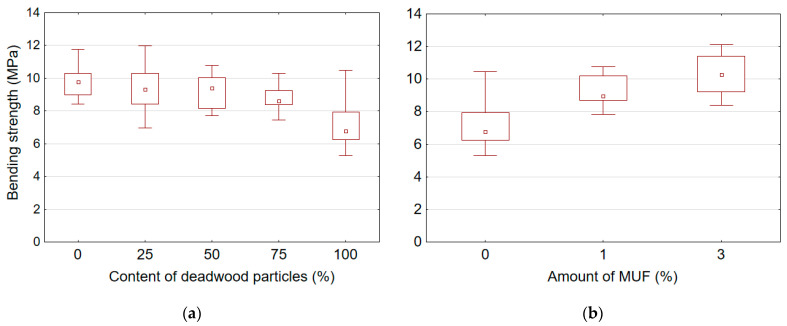
Bending strength of boards samples depending on content of deadwood particles (**a**) and amount of MUF resin in adhesive (**b**).

**Figure 7:**
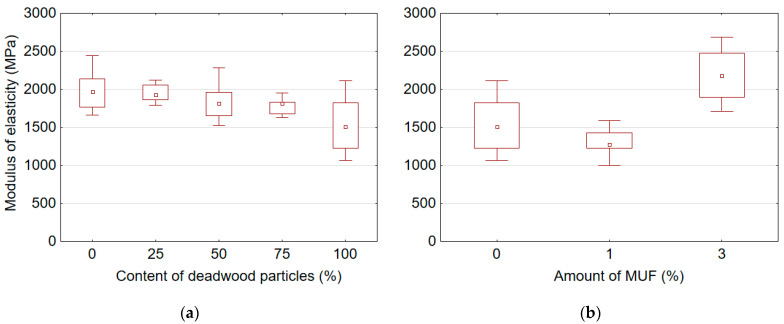
Modulus of elasticity of boards samples depending on content of deadwood particles (**a**) and amount of MUF resin in adhesive (**b**).

**Figure 8:**
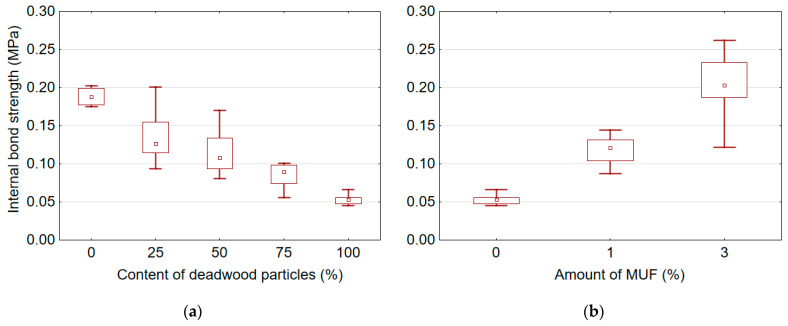
Internal bond strength of board samples depending on the content of deadwood particles (**a**) and amount of MUF resin in adhesive (**b**).

Table 4 presents mean values of mechanical properties for boards manufactured with adding different amount of deadwood particles and various amount of MUF resin into the UF adhesive. It was found that the content of deadwood particles and the amount of MUF resin added into UF adhesive significantly affect the mechanical properties of the boards, including MOR, MOE, and IB. In addition, it was observed that the addition of MUF resin into the UF adhesive has a significantly stronger effect on the mechanical properties than the content of deadwood particles. However, all produced boards did not meet the respective European standard EN-312 requirements [43] (MOR > 11.5 MPa, IB > 0.24 MPa) for applications in dry conditions. To some extent, the low density (≈650 kg/m^3^) of the produced boards can explain this. A graphic representation of the effect of deadwood particle content and amount of MUF resin on the mechanical properties of particleboards is shown in Figure 6, Figure 7 and Figure 8.

It was found that an increase in the deadwood particle content causes a decrease in MOR (Figure 6a), MOE (Figure 7a) and IB (Figure 8a), while an increase in the amount MUF resin, on the contrary, leads to an improvement in these properties. The highest MOR, MOE, and IB values for samples with sound wood particles were 9.75 MPa, 1978.21 MPa, and 0.19 MPa, respectively. The lowest MOR, MOE, and IB values for samples with 100% deadwood particle content were 7.16 MPa, 1521.48 MPa, and 0.05 MPa, respectively. Thus, compared to reference boards made of sound wood particles, the values of MOR, MOE and IB for boards with 100% of deadwood particles are reduced by 26.5%, 23.1% and 72.4%, respectively. The addition of deadwood particles has the strongest effect on the quality of bonding (IB), reducing it by almost four times. The opposite results were obtained by other authors [12,29,30]. Some authors [30] did not find significant differences between particleboard produced from beetle-killed wood and those produced from green wood. Moreover, the composite board made from beetle-killed showed good internal bond test values, acceptable values for MOR and MOE, and a slight increase in thickness swelling and water absorption [12,30]. Adjusting the particle mixture to include 25% material from beetle-killed wood increased both MOR and MOE compared to the 100% green-wood mixture. The water-soak test results were better as well [29].

In general, it was observed that the addition of MUF resin into UF adhesive enables to improve the properties of boards made from 100% of deadwood particles (Figure 6b, Figure 7b and Figure 8b). Already the amount of 1% of MUF resin in the adhesive mix, the MOR of samples made from deadwood particles was higher than that of samples made from sound wood particles. The addition of 3% MUF resin increased MOR, MOE and IB by 44.1%, 43.3%, and 294.4%, respectively.

It can be stated that such changes in MOR for boards from deadwood particles are caused by a greater content of smaller chip fractions (Table 1) in outer layer than for boards from sound wood particles. Due to the large surface area of fine particles, the percentage of its coverage with adhesive is smaller than that of particles of larger fractions. This reduces the total chip bonding area and, as a result, reduces the bending strength of the boards. It is generally known that acceptable wood-based panels require quality particles and the smallest amount of fines, because fines consume excess amounts of resin binder and contribute little to mechanical properties [5].

Significant loss of IB strength (Figure 8a) occurs due to destructive changes in the structure of deadwood, an increased content of small particles fractions in the core layer (Table 1), as well as a large number of cracks that are formed during chipping. The existing adhesive contacts are not enough for strong bonding of particles. One of the reasons for low IB strength is that the wood particles from dead trees were difficult to glue because of apparent surface quality damage due to chipping dry wood [28]. In addition, the presence of a larger number of smaller particles from deadwood (Table 1) also impairs the mechanical properties of the boards. After all, it is well known that as the amount of fines increases, board property values decrease [44]. Moreover, the amount of resin required increases, thereby increasing product manufacturing costs [44]. Study showed that at least 30% more adhesive would be needed to produce commercially acceptable OSB panel products from 100% mountain pine beetle wood; however, such increase of adhesive content is uneconomical [5].

### 3.3. Formaldehyde Release of Boards

**Figure 9:**
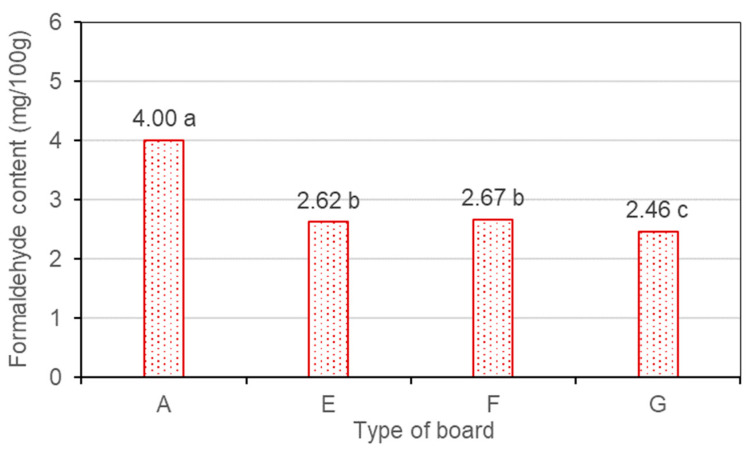
Effect of the amount of MUF resin in adhesive on the formaldehyde content of boards made with wood particles from dead trees. (Averages with the same letter are statistically equal.).

Markedly, the boards (type E) made from deadwood particles like as the control board, reached the E1 emission class (≤8.0 mg/100 g) but characterized by a much lower FC than the reference boards (type A) made from conventional sound wood particles. In the boards made from deadwood particles (type E), the formaldehyde content is lower by 34.5% compared to the reference samples (type A) made from conventional sound wood particles (Figure 9).

Addition of 1% or 3% MUF resin to the UF adhesive did not significantly affect the reduction of formaldehyde content in the boards (F and G). At the maximum added amount 3% of MUF resin, the formaldehyde content in the boards made from deadwood particles decreased by 38.5% compared to the reference boards made from sound wood. Others [45] found that the use of MUF resin increases the formaldehyde emission of the boards from thermo-mechanical and chemo–thermomechanical pulping.

Therefore, we can assume that excessive dryness and low content of extractive substances, as well as long storage time of deadwood, are the factors that led to a decrease in formaldehyde release. It is known that the emission levels of formaldehyde depend on numerous factors such as wood species, moisture content, content of extractives, outside temperature, and storing time [32,34]. The removal of extractives decreases the formaldehyde emitted from the wood. The results reveal that extracted chips release significantly lower amounts of formaldehyde compared to unextracted chips [34]. The air-dried wood produces low emissions of formaldehyde [46].

## 4. Conclusions

This preliminary study confirmed the possibility of manufacturing particleboards using wood particles from deadwood. However, the findings suggest that replacing conventional sound wood particles with deadwood particles leads to deterioration of the physical and mechanical properties of the boards with using UF adhesive. The particleboards from deadwood particles absorb more water and swell more. The MOR, MOE, and IB values for boards with 100% deadwood particles are lower by 26.5%, 23.1%, and 72.4%, respectively, compared to reference boards from sound wood particles. However, the modification of UF adhesive with MUF resin significantly improves the physical and mechanical properties of the boards. Adding 3% of MUF resin to UF adhesive increases MOR, MOE, and IB by 44.1%, 43.3%, and 294.4%, respectively whereas decreases WA 24 h and TS 24 h by 18.2% and 42.9%, respectively. A significant advantage is that boards made from 100% deadwood particles are characterized by 34.5% less formaldehyde content than reference boards made from conventional sound wood.

Further studies on the morphological structure and chemical composition of the deadwood are required, taking into account the age and time since death of trees. These data will help to find out the gluing mechanism and to choose the appropriate adhesive and mode parameters for pressing boards using such wood.

## Figures and Tables

**Table 1:** Fraction analysis (by % weight) of wood particles from deadwood and sound wood.

Outer Layers				Core Layer			
Screen Hole Size (mm)	Content (%)		Difference (±)	Screen Hole Size (mm)	Content (%)		Difference (±)
	Deadwood	Sound Wood			Deadwood	Sound Wood	
1.25	4.42	8.8	−4.38	5.0	9.90	12.0	−2.1
1.0	9.05	1.2	+7.85	3.15	20.69	25.6	−4.91
0.8	12.19	9.4	+2.79	2.0	30.57	31.4	−0.83
0.63	15.24	12.2	+3.04	1.25	24.47	10.6	+13.87
0.4	27.44	26.4	+1.04	0.63	11.78	8.4	+3.38
0.2	19.14	17.6	+1.54	0.32	1.69	1.4	+0.29
Dust	12.52	14.5	−1.98	Dust	0.90	0.6	+0.3
Total	100	100	-	Total	100	100	-

**Table 2:** Manufacturing parameters of particleboards.

Board Type	Content (%) of Wood Particles from		Amount of MUF Resin (%)
	Sound Wood	Deadwood	
A	100	0	0
B	75	25	0
C	50	50	0
D	25	75	0
E	0	100	0
F	0	100	1
G	0	100	3

**Table 3:** Physical properties of particleboards.

Board Type	Density (kg/m^3^)	Water Absorption 2 h (%)	Water Absorption 24 h (%)	Thickness Swelling 2 h (%)	Thickness Swelling 24 h (%)
Effects of wood particles content					
A	630.5 ± 32.1 ab	29.01 ± 8.61 a	90.24 ± 10.05 a	11.03 ± 3.55 a	42.01 ± 6.98 a
B	657.7 ± 28.6 d ^1^	32.59 ± 10.19 ab	90.33 ± 7.68 a	12.49 ± 4.36 ab	45.08 ± 7.20 ab
C	639.3 ± 27.4 bc	37.57 ± 13.31 bc	97.85 ± 8.20 b	16.14 ± 5.30 c	48.55 ± 8.36 bc
D	615.7 ± 70.5 a	35.68 ± 6.27 c	92.18 ± 10.88 a	13.76 ± 4.27 b	41.36 ± 13.00 a
E	649.3 ± 28.6 cd	38.96 ± 9.00 c	99.76 ± 13.31 b	16.17 ± 4.20 c	50.82 ± 7.32 c
Effects of amount of MUF resin					
E	649.3 ± 28.6 ab	38.96 ± 9.00 b	99.76 ± 13.31 b	16.17 ± 4.20 c	50.82 ± 7.32 c
F	656.9 ± 32.7 b	36.39 ± 11.59 b	96.07 ± 9.10 b	12.31 ± 3.34 b	38.63 ± 3.41 b
G	638.4 ±32.8 a	25.29 ± 7.78 a	81.63 ± 7.86 a	7.73 ± 2.25 a	29.00 ± 3.15 a

^1^ Averages followed by the same letter at the column are statistically equal by the Duncan test at 95% probability.

**Table 4:** Mechanical properties of particleboards.

Board Type	MOR (MPa)	MOE (MPa)	IB (MPa)
Effects of wood particles content			
A	9.75 ± 0.97 b ^1^	1978.21 ± 257.63 c	0.19 ± 0.02 e
B	9.33 ± 1.48 b	1928.68 ± 157.98 bc	0.14 ± 0.03 d
C	9.17 ± 1.00 b	1838.84 ± 247.09 bc	0.12 ± 0.03 c
D	8.72 ± 0.96 b	1747.08 ± 189.10 b	0.08 ± 0.02 b
E	7.16 ± 1.49 a	1521.48 ± 341.64 a	0.05 ± 0.01 a
Effects of amount of MUF resin			
E	7.16 ± 1.49 a	1521.48 ± 341.64 a	0.05 ± 0.01 a
F	9.25 ± 0.95 b	1329.41 ± 213.61 a	0.12 ± 0.02 b
G	10.32 ± 1.24 c	2180.54 ± 328.35 b	0.21 ± 0.03 c

^1^ Averages followed by the same letter at the column are statistically equal by the Duncan test at 95% probability.

## Data Availability

Not applicable.
